# Duodenal Leishmaniasis Mimicking Celiac Disease in HIV Patient: A Case Report and Literature Review

**DOI:** 10.7759/cureus.60153

**Published:** 2024-05-12

**Authors:** José Vitor Santos-Oliveira, Gabriella Vanin, Rosely Antunes Patzina, Richard Calanca, Jose C Ardengh

**Affiliations:** 1 Medicine, Institute of Infectious Diseases Emilio Ribas, São Paulo, BRA; 2 Pathology, São Paulo University, São Paulo, BRA; 3 Endoscopy, Institute of Infectious Diseases Emilio Ribas, São Paulo, BRA; 4 Gastrointestinal Endoscopy, Hospital das Clínicas de Ribeirão Preto, Ribeirão Preto, BRA; 5 Imaging Diagnostic, São Paulo Federal University, São Paulo, BRA

**Keywords:** diferencial diagnosis, duodenal biopsy, upper endoscopy, hiv aids, celiac disease and autoimmunity, visceral leishmaniasis (vl)

## Abstract

It is known that there are several clinical forms that diseases can take when presented in patients living with HIV, especially those in the AIDS phase. Here, we present a case that demonstrates the peculiar capacity of diseases to assume the most varied forms, highlighting the limited research on neglected infectious parasitic diseases. This study aimed to underscore the ability of these diseases to mimic other pathologies, emphasizing the importance of infectious diseases as differential diagnoses in the most diverse clinical entities, as is the case of visceral leishmaniasis.

## Introduction

Visceral leishmaniasis (VL) is a zoonosis of tropical areas and endemic in Brazil [1-3]. It is a disease with important clinical and epidemiological relevance worldwide, with an incidence of approximately 500,000 cases per year. Although neglected, it is considered by the WHO one of the seven global endemics with absolute priority for its mapping [4,5]. Transmission occurs through the bite of the *Lutzomyia longipalpis* mosquito and controlling this vector is also an essential part of eradicating the disease [6-9].

The duodenal form of this parasitosis occurs in about 5% of cases, being even more relevant in patients with compromised cellular immunity, such as in HIV/AIDS patients [3,10]. Despite being endemic, its diagnosis is often delayed, facilitating the spread of the disease and enabling the development of its severe form [10]. The most common clinical manifestations of VL are fever, asthenia, weight loss, and splenomegaly [11].

It is known that VL with duodenal involvement resembles other diseases of the gastrointestinal tract, such as Whipple's disease, autoimmune inflammatory diseases, and celiac disease with similar clinical and histological pictures, making its diagnosis difficult when the level of clinical suspicion is low [10]. In the literature, we found only one case of a patient with VL without HIV, mimicking celiac disease, whose diagnosis was made by duodenal biopsy, through the detection of the intracellular parasite in the pathological examination [12].

For this reason, the authors thought it important to describe the endoscopic findings and diagnostic form of a patient with confirmed HIV-associated kala-azar with previous hospitalizations, being able to search for the parasite in the duodenum due to endoscopic findings similar to celiac disease.

## Case presentation

The patient is a 29-year-old male living with HIV (people living with HIV {PLHIV}) diagnosed seven years ago. He was treated erratically for long periods without antiretroviral medication. One year and eight months ago, he was diagnosed with VL and treated with liposomal amphotericin B (28 in-hospital doses + complementary doses for secondary prophylaxis). Ten months after treatment, he was hospitalized with hyporexia, asthenia, weight loss, intermittent night fever, and upper abdominal pain. On physical examination, he was in good general condition, emaciated, and hypochoric. Hepatosplenomegaly was identified on abdominal palpation.

**Figure 1:**
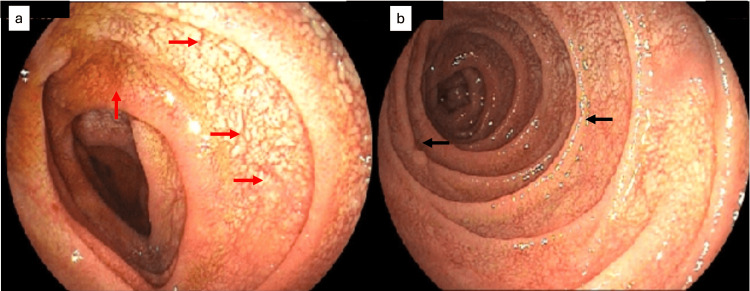
Irregular, nodulariform duodenal mucosa (red arrows) with slightly whitish roughness and fissures (black arrows).

A new diagnosis of VL was made by immunology and a blood test for *Leishmania infantum*. The myelogram test was done to evaluate the bone marrow, as the bone marrow is located inside the bones, the myelogram was performed through a bone puncture of the iliac bone, followed by aspiration of the bone marrow, which was performed under local anesthesia. This test showed amastigotes forms of the intracellular parasite. During hospitalization, he received amphotericin B deoxycholate for six days and then liposomal amphotericin B 300 mg/day, without completing the treatment, by hospital evasion. Ten months later, the patient returns with similar complaints as before, associated with nausea and diarrhea. The diagnosis of VL was again confirmed. At this time, he had a CD4 count of 124/mm^3^ and an undetectable viral load. His upper digestive endoscopy (EDA) showed irregular, nodulariform duodenal mucosa, with roughness and sometimes slightly whitish fissures, with endoscopic features that favored celiac disease (Figures 1a, 1b).

**Figure 2:**
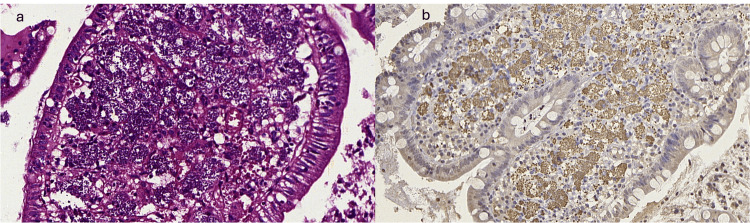
H&E with histiocytic infiltrate containing leishmania amastigotes (400×) (a). Positive immunohistochemistry for leishmania (200×) (b).

The pathological analysis of the duodenal biopsies revealed a dense histiocytic infiltrate in the lamina propria of the duodenal villi (Figure 2a). The histiocytes contained ovoid structures that were revealed to be Leishmania amastigotes on Giemsa staining. Immunohistochemical research was also performed to confirm the diagnosis (Figure 2b).

New treatment with liposomal amphotericin B 300 mg/day was performed for nine days and the cumulative dose was 2700 mg - 38 mg/kg total, in immunocompromised patients, the total dose of liposomal amphotericin B needs to be increased, reaching a value close to 40 mg/kg as happened to our patient [13]. After treatment, the patient progressed with improvement of symptoms, cessation of nausea and diarrhea, and reduction of visceromegaly, without any other adverse event. Twelve days later he was discharged from the hospital and referred to outpatient follow-up for administration of liposomal amphotericin B. The patient received weekly doses for four weeks. During the period of secondary prophylaxis, a dose was given every 21 or 28 days until the patient had a TCD4 lymphocyte count >350 cells.

## Discussion

VL is a parasitic disease whose etiological agent is parasites of the Leishmania species with two of them with greater clinical relevance, *L. donovani *and *L. infantum* [5]. It is an endemic disease in several regions of the world, especially in the Americas, Asia, and Africa. There are estimates that about 90% of cases of VL occur in five following countries: India, Bangladesh, Sudan, South Sudan, Brazil and Ethiopia [4].

With the advance of the AIDS epidemic, the incidence of cases of coinfection between Leishmaniasis and HIV has become increasingly frequent [10]. It is extremely necessary that this population be known, since coinfection with HIV increases the lethality and relapse rate of visceral leishmaniasis exponentially [1]. When not treated, VL has a very high mortality rate, affecting almost the totality of cases [4].

In immunosuppressed patients, besides the greater difficulty of treatment and greater chance of recurrence, another point of extreme importance in clinical practice is the anomalous forms of presentation of VL. In general, leishmania has a predilection for some organs, such as liver, spleen, and bone marrow; however, in people living with HIV, the manifestation of the disease is more frequently seen in structures that are not part of the reticuloendothelial system [1,2,12,14].

One of the systems that can eventually be affected by VL in patients with immune deficiency is the digestive system. The form of presentation of VL-HIV coinfection can be extremely varied. The most common form of presentation is an exacerbation of the classic signs and symptoms, such as persistent fever, adynamia, weight loss, hepatosplenomegaly, and pancytopenia. However, in special situations, such as in parasitism of the gastrointestinal tract, other commemoratives may be added to the patient's history, such as the occurrence of diarrhea [1,9,12].

In the case reported here, our patient had intense duodenal parasitism leading to systemic repercussions, such as diarrhea, fever, weight loss, resembling a dysabsortive syndrome, in addition to alteration of the standard morphology of the affected area, simulating the clinical and endoscopic appearance of celiac disease. In the literature, there are some reports of other pathologies that have been mimicked by VL, such as Whipple's disease and even autoimmune inflammatory bowel diseases [9,15,16].

Given its ability to mimic and variable clinical presentation, the diagnosis of VL may be difficult, especially in those cases where the patient has no previous history of infection and does not reside in a region endemic for this disease. Thus, it is evident that direct research tests, such as upper digestive endoscopy associated with biopsy, can aid in the diagnosis and understanding of the disease progression [16]. However, it is worth noting that despite being extremely prevalent in several regions of the world, this pathology continues to be neglected and consequently kept away from the clinical reasoning of many physicians, so the authors found it interesting to discuss the endoscopic findings of this particular case [4].

## Conclusions

This case showed an EDA with an unusual macroscopic appearance in a patient living with HIV and parasitized by leishmaniasis, mimicking the presentation of celiac disease. Thus, we draw attention to the need to expand the studies on this coinfection, showing the spectrums of severity and the various clinical pictures that this pathology can present, besides demonstrating the broad field of possibility for research in this area.
